# Mechanism of online emotional support accompany group for stress: The role of social support

**DOI:** 10.3389/fpsyg.2022.1047364

**Published:** 2023-01-16

**Authors:** Yingjun Zhang, Heliang Huang, Daisheng Tang, Xiaohua Lu, Fumin Fan, Jingyi Pan

**Affiliations:** ^1^Psychological Education and Counseling Center, Beijing Normal University, Beijing, China; ^2^Faculty of Psychology, Beijing Normal University, Beijing, China; ^3^School of Economics and Management, Beijing Jiaotong University, Beijing, China; ^4^Counseling Center, Beijing Jiaotong University, Beijing, China; ^5^Department of Psychology, Tsinghua University, Beijing, China

**Keywords:** Online Emotional Support Accompany Group, stress, social support, shame, loneliness, COVID-19

## Abstract

**Objective:**

To investigate the effect of social support on stress, and to clarify the effect and mechanism of Online Emotional Support Accompany Group (OESAG).

**Methods:**

The group members who signed up for the public welfare project “Psychological Rehabilitation Group Psychological Service under the COVID-19 Pandemic” were divided into the treatment group, the control group, and the blank group with 37 members each. The treatment group received OESAG intervention, the control group received online time management group intervention, and the blank group was the waiting group. The three groups of subjects were synchronously tested before and after the intervention group.

**Results:**

After the OESAG intervention, compared with the control group and the blank group, the treatment group showed that perceived social support was improved, and loneliness and stress were decreased.

**Conclusion:**

Improving social support can effectively reduce stress. OESAG can effectively improve social support and so too decrease stress. This study could help in designing effective psychological intervention measures to reduce the degree of stress symptoms and enhance both personal and social levels of coping with stressful events.

## Introduction

In late 2019, the coronavirus disease 2019 (COVID-19) pandemic began to spread around the world, triggering the worst public health incident in recent years. Since the outbreak of COVID-19, daily life of the public has suffered a huge shock. COVID-19 threatens people’s safety not only physically but also psychologically (Ahmed et al., 2020). One of the most obvious is the psychological stress response due to COVID-19. Panic about the pandemic, incompatibility with pandemic prevention measures such as control and quarantine, and changes in social relations during the pandemic all make individuals prone to stress reactions (Taylor et al., 2020; Zhou et al., 2020).

## Literature review

### Stress under COVID-19

Stress was proposed by Selye (1936) and refers to the non-specific response of the body to any changing demands. The COVID-19 crisis has greatly affected human lives across the world. Uncertainty and quarantine have been affecting the mental health of people. The prevalence rate of all forms of depression, anxiety, stress, sleep problems, and psychological distress in the general population was found to be higher during the COVID-19 pandemic (Lakhan et al., 2020; Li et al., 2020; Marmarosh et al., 2020).

Among the people affected by the COVID-19 pandemic, psychological intervention was very important (Zhong et al., 2020), but due to the time and space constraints caused by the pandemic, traditional face-to-face psychological counseling intervention was difficult to carry out. In addition, the current individual psychological counseling reserve cannot meet the huge demand of the public for psychological intervention. In contrast, online group psychological intervention can be more efficient and without space limitation. During the pandemic, a study proved that a single online intervention was able to reduce depression symptoms in adolescents under pandemic (Schleider et al., 2022). In a study of chronic patients, online single-intervention groups were found to reduce negative emotions such as pain and anxiety, and depression in chronic patients (Ziadni et al., 2021). A study found that a single online group psychological counseling session can effectively provide psychological support for the public and relieve psychological pressure under COVID-19 (Ni et al., 2022). These related studies all showed that a single online group intervention can reduce the level of negative emotions and provide psychological support for the participants.

Therefore, it is feasible and necessary to develop a new and effective online group psychotherapy model to intervene stress.

### Online group intervention

Online interventions are effective in coping with the mental health problem of people, as experienced due to the pandemic, and in reducing symptoms of anxiety, depression, and stress (e.g., Situmorang, 2020; Shapira et al., 2021; Zhang et al., 2021; Silva et al., 2022).

However, there is still lots of controversy about online groups in academic circles. Compared with traditional counseling or therapy, online groups face many challenges, such as confidentiality, safety, effectiveness, etc. (Weinberg, 2020). The COVID-19 outbreak has caused a demand for psychological intervention (Wang et al., 2020). At the same time, pandemic prevention requirements such as home quarantine and social distancing have also resulted in physical space isolation. Therefore, the online intervention has been identified as a useful tool in providing psychological assistance and support (Carbone et al., 2022).

Scholars who have undertaken research in response to the pandemic have filled the gap in this area by conducting studies on the effects of online group psychological counseling during the pandemic (Xiong et al., 2022; Situmorang, 2022; Urkmez et al., 2021), all confirmed the intervention effect of online group psychological counseling.

### Online emotional support accompany group

The psychological stress response caused by the pandemic has generally affected the lives of people (Taylor et al., 2020). Data from China, for example, suggests that 25% of the general population has experienced moderate to severe levels of stress- or anxiety-related symptoms in response to COVID-19 (Qiu et al., 2020; Wang et al., 2020). Empirical research shows that group intervention has positive effects on stress response (Park and Kim, 2021; Mumba et al., 2022). Pre-COVID-19, various scholars proposed intervention models for stress from different intervention perspectives. For example, Brymer et al. (2006) proposed in *Psychological first aid: field operations guide, 2nd edn.* That the intervention goals include providing emotional comfort, processing emotions, providing information, and establishing links with social support networks, etc. Deng (2010) believed that the core of the intervention method was to “listen to their voices attentively and accompany them to solve problems.”

The book written by Deng (2010) *Handbook of Crisis Counseling and Psychological First Aid for Disaster* is classic and effective in-tervention guidance for disaster crisis scenarios. For this reason, project members related to the current study received training in the program during the pandemic. Because of the quarantine policy, the Internet platform became the common communication method. Brouzos et al. (2021a) verified the effect of online positive group psychological intervention for people during the pandemic. What was needed was a short-term intervention. Therefore, the project team designed a single-session Online Emotional Support Accompany Group (OESAG) according to the intervention goals and methods of previous researchers (Deng, 2010; Brouzos et al., 2021a; Lu et al., 2022a). OESAG is a semi-structured single-session group. Under the facilitation of the group leader, it creates a safe online interpersonal space to promote group members to connect, hear each other, see each other, gain social support, transform emotions, and gain strength. OESAG targets the three important influencing factors in previous studies which are perceived social support, loneliness, and shame (Lu et al., 2022a). It aims to reduce stress responses by intervening in these targets. Therefore, based on the results of previous studies exploring the relationship between social support and stress, this study uses OESAG to manipulate the social support and observe changes in stress response to verify the role of social support on stress response and its mechanism. Hypothesis 1 of this study: OESAG can reduce stress related to the pandemic.

#### Social support

Social support is considered to be an important factor in being able to cope with mental health problems (Toplu-Demirtas et al., 2018; Wang et al., 2018). A study during the COVID-19 pandemic found that increased social support is significantly correlated with decreased psychological distress, and may serve as the basis for psychological interventions (Huang and Zhang, 2022). The social support buffer model was proposed by Cohen and Wills (1985). According to the social support buffer model, social support is a resource used by individuals to cope with stressful events. Individuals can feel the improvement of self-worth through social support, thereby inhibiting or buffering the negative impact of stressful events on individual emotions. The findings of a study on psychosocial support in Chinese during the COVID-19 pandemic suggest that social support buffers the negative effects of loneliness in the context of the perceived severity of COVID-19 (Wang et al., 2022). A study of school students found that when faced with stress, subjects who perceived higher levels of social support experienced less stress (McLean et al., 2022). A study provides evidence of the buffering effect of social support for the association between stress and the response spectrum of the hypothalamic–pituitary–adrenal (HPA) axis (Chen et al., 2021). HPA axis hyperactivity owing to psychosocial stress has been proposed as a potential pathway underlying the link between social support and health (Iob et al., 2018). The results of a study on the relationship between social support and the physical function and physical health of employees show that social support at work can have a profound impact on the physical health of employees (Gonzalez-Mulé and Yuan, 2022).

Based on the above theory, this paper proposes Hypothesis 2: OESAG group intervention can enhance social support.

#### Loneliness

China has adopted quarantine measures for pandemic prevention. Quarantine means the loss of connection between people (Zhong et al., 2020). Therefore, during the pandemic, loneliness may be the most obvious negative emotion experienced by individuals. During the pandemic, this kind of loneliness is the main factor that threatens the physical and mental health of people (Banerjee and Rai, 2020). Loneliness can affect health, with one study linking loneliness to increased mortality and an increased risk of certain cardiovascular, metabolic and neurological diseases (Hawkley, 2022).

A study found that stress response may be a biological mechanism of loneliness affecting health (Brown et al., 2018). In a study of Arabs in Israel during the COVID-19 pandemic, loneliness levels were found to positively predict stress levels (Ali-Saleh and Halperin, 2022). During the pandemic, measures, such as reducing gatherings, regional control, and maintaining social distancing have reduced the connection and support between individuals, increased individual loneliness, and may make individuals more prone to stress reactions (Hwang et al., 2020).

#### Loneliness and social support

Social support is an important coping resource for individuals to face negative emotions and an important buffer factor for loneliness. There is a great deal of current research on the ability of social support among older adults to effectively reduce loneliness. Studies have shown that increased social support among older adults can effectively reduce loneliness (Kang et al., 2018; Han et al., 2021; Wu, 2022). Interventions enhanced the perceived social support of participants can reduce loneliness effectively during the pandemic (Bareket-Bojmel et al., 2021). Yildiz and Duyan (2022) found that group counseling can reduce loneliness. Also, group counseling can improve social interaction and alleviate the interpersonal experience of loneliness (Hidayati et al., 2022). Therefore, this paper proposes Hypothesis 3: OESAG group intervention can effectively reduce loneliness.

#### Shame

During the COVID-19 crisis, shame has become an important emotion with a powerful effect (Berry, 2020). Shame is an acute stress response to interpersonal traumatization (Trumbull, 2020). In public health events caused by infectious diseases, people often feel ashamed for having contracted or transmitted the virus. During the pandemic, the fear of being accused of spreading the disease or of being exposed to the public made it easier for people to feel ashamed, and the rapid increase in the efficiency of information dissemination (social media, We Media, etc.) somehow magnifies this shame (Cavalera, 2020). Shame is a state in which cognition is influenced by social interactions and negative emotions about oneself, resulting from the belief of an individual that a person has failed to meet social expectations, or that he or she experienced social threats (Woodward, 2021; Lu et al., 2022b).

#### Shame and social support

Social support can help individuals cope with negative emotions such as buffering shame. In some studies, it has been found that shame and social support are negatively correlated, and the higher the social support, the lower the shame (Oliser, 2021; Lu et al., 2022b). A study suggests social support may be an effective coping strategy for psychological distress during the COVID-19 pandemic (Marmarosh et al., 2020; Rathakrishnan et al., 2022). In another study, it was found that perceived social support reduced individual stigma, and therefore personal shame (Broman et al., 2022).

Therefore, this paper proposes Hypothesis 4: OESAG group intervention can effectively reduce shame.

### The current study

Under the premise of urgent social needs, OESAG was developed to alleviate stress and also predict the ability to cope with stress caused by the pandemic. The study aimed to find empirical evidence to prove that OESAG can reduce stress and that OESAG can enhance social support which was the main target of it. Furthermore, the study also investigated whether OESAG can effectively reduce loneliness and shame which were important effects close to stress under the pandemic.

## Materials and methods

### Participants

From March to June 2020, we used Public WeChat to spread the brochure in collaboration with hospitals, the Public Security Bureau and the community. Potential group members could sign up for OESAG and time management groups (Zhang, 2021) and fill out the questionnaires *via* the brochure. We randomly selected eight OESAG groups and two time management groups, the control group, and the waiting group [set as the blank group; Enrollment criteria: voluntary registration for online group psychological counseling. Exclusion criteria: (1) those with a psychiatric diagnosis; (2) those with interpersonal communication disorders; (3) Those who are infected or suspected of being infected with COVID-19 or, their family members, relatives, and friends]. All the groups were conducted *via* Zoom (online). Ethical approval was given by Tsinghua University; the ID is [(2021) Ethical Approval. NO.23].

Finally, 37 participants were obtained for each group (treatment group, control group, and blank group). Multiple online groups included 8–20 participants per group per component. The treatment group was given OESAG intervention, the control group was given online time management group intervention, and the blank group was the waiting group (entering the registered group after completing the posttest). Three groups of subjects were tested before and after the same period (pretest before the start of the group and posttest 0–48 h after the end of the group). Three groups were tested by the administrator of the project before and after groups *via* WeChat groups.

There was no significant difference in the scores of the pretest stress perception scale between the treatment group (25.34 ± 5.97), the control group (25.41 ± 4.87), and the blank group (24.44 ± 6.59). Most of the participants were 31–40 years old (51.4, 67.6, and 64.9% in treatment, control, and blank group), and had higher education (94.6, 43.3, and 97.3% had postgraduate and higher education in treatment, control and blank group). There were no significant differences in age, gender, education, and marital status among the three groups, as shown in Table 1.

**Table 1:** Demographic data comparison for three groups.

	Classification	Treatment group(*N* = 37)	Control group(*N* = 37)	Blank group(*N* = 37)	χ^2^	*p*
Age	18–30	12(32.4%)	8(32.6%)	10(27.0%)	20.43	<0.001
	31–40	19(51.4%)	25(67.6%)	24(64.9%)		
	41–50	3(8.1%)	1(2.7%)	2(5.4%)		
	51~	3(8.1%)	3(8.1%)	1(2.7%)		
Gender	Female	33(89.2%)	34(91.9)	29(78.3%)	3.23	0.198
	Male	4(10.8%)	3(8.1%)	8(21.6%)		
Marriage	Single	6(16.2%)	3(8.1%)	6(16.2%)	1.38	0.500
	Married	31(83.8%)	34(92.9%)	31(83.8%)		
Education	Specialty and lower	0	2(5.4%)	0	25.3	<0.001
	Undergraduate	2(5.4%)	9(24.3%)	1(2.7%)		
	Postgraduate and higher	35(94.6%)	16(43.3%)	36(97.3%)		

The data in this study were all collected from the psychological counseling projects of public welfare groups carried out during the psychological assistance stage of the COVID-19 pandemic. All the subjects were openly recruited people who participated in the psychological counseling of public welfare groups. The study was ethically approved and was approved by the group counseling project team and supported by crisis intervention experts.

### Measures

#### Perceived stress scale

Perceived Stress Scale (PSS) measured stress responses, was developed by Cohen et al. (1994) and was revised by Wang et al. (2015) into Chinese version. A total of 10 items are divided into two dimensions: negative feelings and positive emotions. The scale has shown good reliability and validity among Chinese people (Wang et al., 2015; Lu et al., 2022b). Sample items include “in the last month, how often have you been upset because of something that happened unexpectedly?” and “In the last month, how often have you felt confident about your ability to handle your personal problems?” A five-point scale was used (1 = strongly disagree to 5 = strongly agree), with higher scores indicating more pronounced stress. Cronbach’s α of this questionnaire in this study was 0.82–0.84, the test/retest reliability was 0.72 and the KMO value was 0.89 (*p* < 0.001) which showed acceptable validity.

#### Loneliness scale

Loneliness scale (UCLA Loneliness Scale, UCLA) was used to assess loneliness. UCLA was compiled by Russell et al. (1980) had its Chinese version by Wang et al. (2019), which has a total of 20 items. The Chinese version has good reliability and validity among Chinese people and is widely used (Wang et al., 2019; Han et al., 2021; Lu et al., 2022b). Each item reflects the individual’s relationship state and relevant inner feeling (e.g., “I feel in tune with the people around me,” “I lack companionship”). It is a four-point scale (1 = never to 4 = always), the higher the score, the higher the degree of loneliness. Cronbach’s α of this questionnaire in this study was 0.79, the test/retest reliability was 0.73 and the KMO value was 0.91 (*p* < 0.001) which showed sufficient validity.

#### Shame scale

The Shame Scale (SS) measured shame. SS was prepared by Qian et al. (2000) in Chinese. SS has satisfying reliability and validity and is widely used in shame study in china. Sample items like “Are you ashamed of certain personal behaviors?,” “Are you ashamed of your ability to do things?” Twenty-five items were selected, including the three dimensions of personality shame, behavioral shame, and family shame. It is a four-point scale (1 = never to 4 = always), the higher the score, the higher the degree of shame. In this study, Cronbach’s α of this questionnaire was 0.85, the test/retest reliability was 0.72, and the KMO value was 0.96 (*p* < 0.001) which showed adequate validity.

#### Social support scale

The Perceived Social Support Scale (PSSS) was used to assess social support. PSSS was compiled by Zimet et al. (1988) and used by Jiang (1999) (as Chinese version). The Chinese version of PSSS has satisfying reliability and validity and is widely used among Chinese participants (Jiang, 1999; Chen et al., 2021; Lu et al., 2022b). Each item reflects the individual’s subjective interpersonal resources [i.e., “When I encounter problems, some people (leaders, relatives, and colleagues) will appear beside me.”]. Totally, 12 items, a seven-point scale (1 = strongly disagree to 7 = strongly agree), the higher the score, the higher the overall degree of social support the individual feels. Cronbach’s α of this questionnaire in this study was 0.93, the test/retest reliability was 0.76, and the KMO value was 0.92 (*p* < 0.001) which showed sufficient validity.

### Design of experiments

This study adopted 3 (treatment group, control group, blank group) × 2 (pre- and post-test) experimental design.

## Procedure

### Group plan and implementation

Online Emotional Support Accompany Group is a single online group psychological intervention model developed based on the goals and methods of stress intervention proposed by Brymer et al. (2006) and Deng (2010) OESAG takes a total of 90 min and includes five steps. The first step: Relationship building. Let the members get to know each other through self-introduction of the leader and members. The second step: Boundary setting. Leaders introduce group goals, and principles and create a safe group atmosphere. The third step: Express freely. The members talked about their life experiences in the past month in turn. The fourth step: Supportive feedback. Members provide supportive feedback on the part of the group that embodies the resource. For example, “I appreciate the bravery of × × ×.” The fifth step: Conclude and farewell. Members reflect on the group experience, summarize the gains in one sentence, and say goodbye to the group.

The Time-Management Group has also created during this project. The plan includes five steps: Warm-up, life changes under the pandemic, life running account, time pies, summary, and farewell, a total of 90 min (Zhang, 2021).

As the Time-Management Group has a similar length and also works with a group model, it was used as the control group to compare with OESAG.

All groups use a co-leader model (Yalom and Leszcz, 2020). All leaders received basic training in group psychological counseling and also received normative training about the corresponding group model, and are all led under the supervision of registered supervisors of the Chinese Psychological Society.

## Results and analysis

### Results

**Figure 1:**
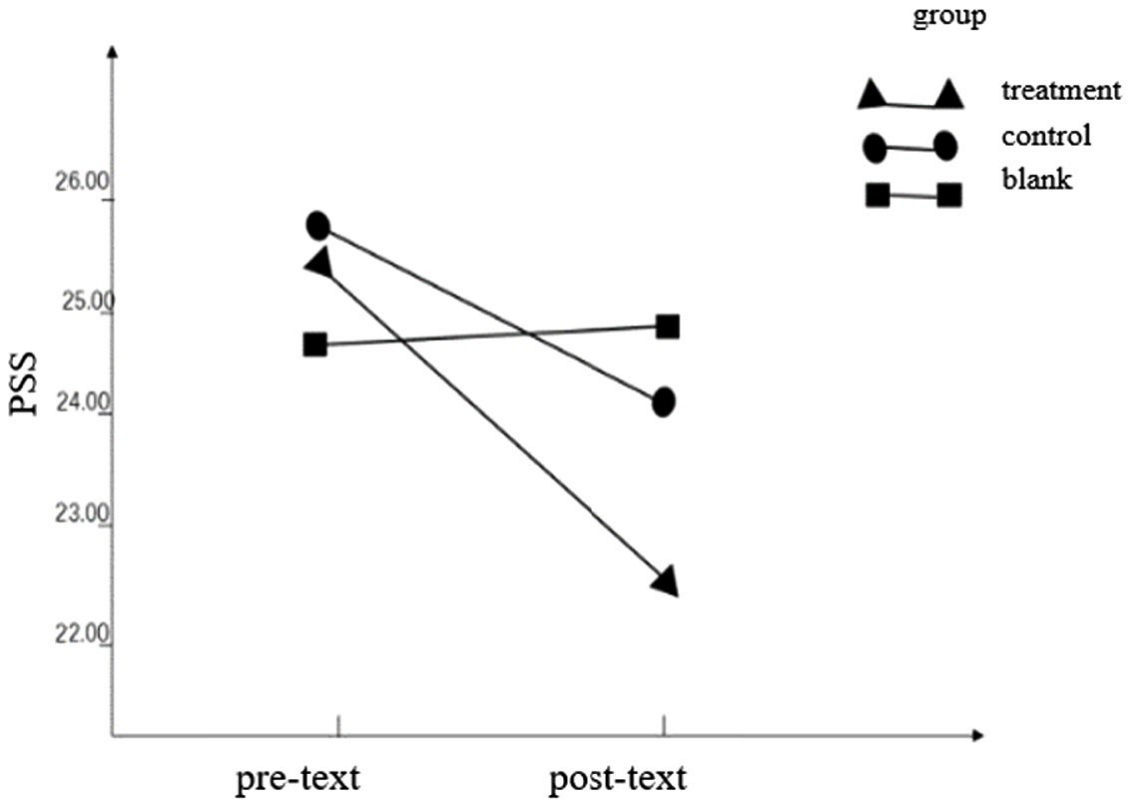
Pre- and post-text outcome of PSS.

Using SPSS 22.0 (Version 22.0, IBM, United States) to conduct a two-way repeated measures ANOVA, it was found that on the PSS score, the interaction between grouping and time point was statistically significant (*F* = 6.40, *p* = 0.017, η_p_^2^ = 0.171), indicating that different groups were in the front and back. The perceived stress scale scores were different. The main effect of the group was not statistically significant (*F* = 0.212, *p* = 0.648, η_p_^2^ = 0.007). The time-point main effect was statistically significant (*F* = 14.112, *p* < 0.001, η_p_^2^ = 0.313). Simple effects analysis showed that the pretest score of the treatment group (25.34 ± 5.97) was higher than that of the posttest (22.56 ± 6.37), and the difference between the control group and blank group was not statistically significant (25.41 ± 4.87 vs. 24.03 ± 5.09; 24.44 ± 6.59 vs. 24.72 ± 6.74), indicating that the perceived stress of participants after OESAG intervention was reduced, as shown in Table 2; Figure 1.

**Table 2:** Pre- and post-test outcomes.

	Group	Pretest*M* (*SD*)	Post-test*M* (*SD*)	*F*	*post hoc* test *p* < 0.05
PSS	Treatment group	25.34 (5.97)1	22.56 (6.37)2	6.40 ^**^	1 > 2
	Control group	25.41 (4.87)3	24.03 (5.09)4		
	Blank group	24.44 (6.59)5	24.72 (6.74)6		
SS	Treatment group	56.70 (12.06)1	52.73 (14.122)	0.62	
	Control group	57.89 (17.14)3	52.38 (17.04)4		
	Blank group	51.81 (14.82)5	50.41 (16.99)6		
UCLA	Treatment group	44.97(8.42)1	37.94(8.64)2	4.70 ^*^	1 > 2
	Control group	42.08 (7.08)3	40.22 (10.12)4		
	Blank group	37.86(8.50)5	40.56(9.74)6		
PSSS	Treatment group	49.35 (11.33)1	59.46 (10.26)2	18.34 ^**^	1 > 2; 5 > 1; 5 > 3
	Control group	50.81 (11.93)3	58.97(15.72)4		
	Blank group	62.32 (13.32)5	58.68 (17.49)6		

^*^p < 0.05, ^**^p < 0.01.

**Figure 2:**
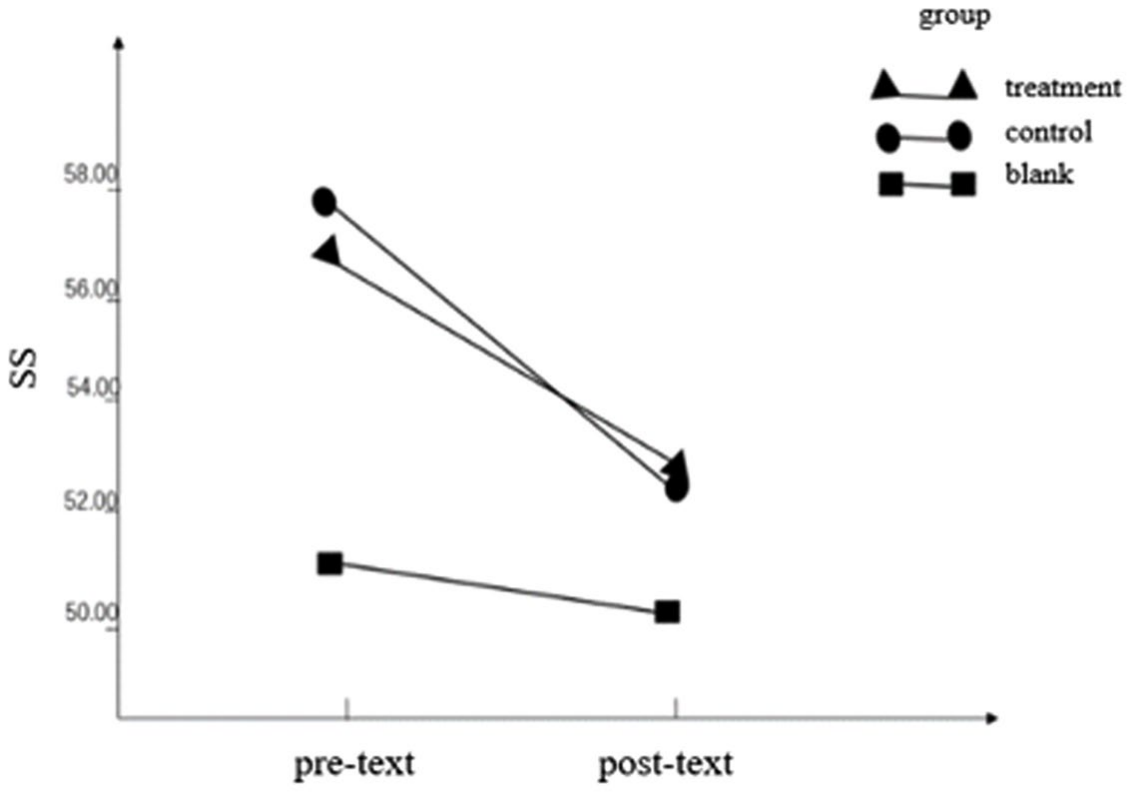
Pre- and post-text outcome of SS.

On the SS score, the interaction between the group and time point was not statistically significant (*F* = 0.62, *p* = 0.436, η_p_^2^ = 0.017). The main effect of the group was not statistically significant (*F* = 1.355, *p* = 0.252, η_p_^2^ = 0.036). The main effect of the time point was statistically significant (*F* = 8.267, *p* = 0.007, η_p_^2^ = 0.187), and the pretest was 55.47 ± 1.56, which was significantly higher than the posttest (51.84 ± 1.47), indicating that the shame of the participants over time, as shown in Table 2; Figure 2.

**Figure 3:**
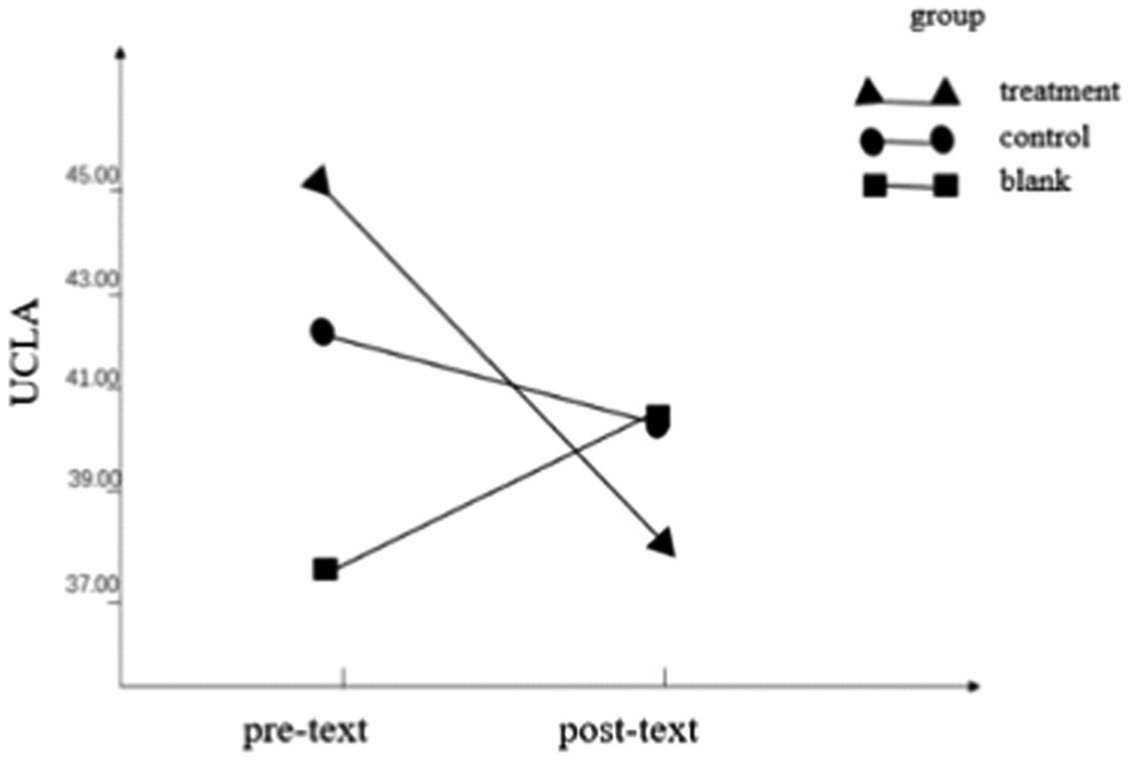
Pre- and post-text outcome of UCLA.

Regarding the UCLA score, the interaction between the group and time point was statistically significant (*F* = 4.70, *p* = 0.037, η_p_^2^ = 0.118), indicating that the loneliness scores of different groups were different before and after the test. The group main effect (*F* = 2.38, *p* = 0.132, η_p_^2^ = 0.064) and the pre- and post-test main effect (*F* = 3.001, *p =* 0.092, η_p_^2^ = 0.243) were not statistically significant. Simple effects analysis found that the pretest score (44.97 ± 8.42) of the treatment group was higher than the posttest score (37.94 ± 9.64), indicating that the loneliness of the subjects after the OESAG intervention was reduced, as shown in Table 2; Figure 3.

**Figure 4:**
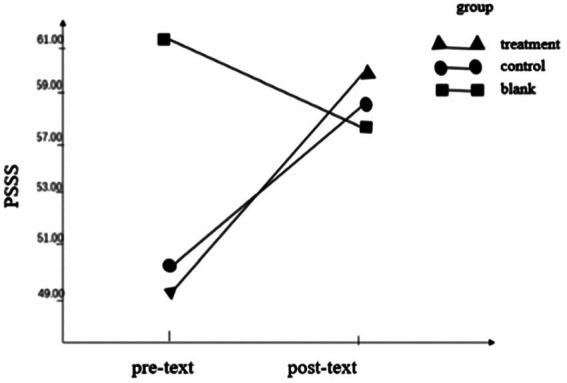
Pre- and post-text outcome of PSSS.

Regarding the perceived social support score, the interaction between the group and time point was statistically significant, *F* = 18.34, *p* < 0.001, η_p_^2^ = 0.338, indicating that the perceived social support of different groups was different before and after the test. The group main effect (*F* = 11.53, *p* = 0.002, η_p2_ = 0.303) was statistically significant with the time point main effect (*F* = *8.87*, *p* = 0.005, η_p2_ = 0.198). Simple effects analysis found that the pretest score (49.35 ± 11.33) of the treatment group was higher than that of the posttest (59.46 ± 10.26), indicating that the perceived social support of the participants after the OESAG intervention was improved, as shown in Table 2; Figure 4.

## Discussion and conclusion

### Discussion

Compared with the control group and the blank group, the OESAG intervention group increased social support and decreased stress, which is consistent with hypotheses 1 and 2, and a large number of practitioners observed that individually perceived social support helps to reduce stress (e.g., Deng, 2010; Brouzos et al., 2021b; Lu et al., 2022a; Marmarosh et al., 2020). OESAG is a form of a single-session group intervention for coping with the psychological stress of the COVID-19 pandemic. Its purpose is to provide social support and enhance individual perceived social support, thereby reducing stress. Social support is an important coping resource for individuals and has a generally beneficial effect on maintaining individual mental health, as the acknowledged social support buffer model noted, so it has a stabilizing effect on coping with stress. Therefore, during the pandemic, people are in a state of stress such as panic, anxiety, and depression. A supportive and connected environment will probably help to alleviate stress during the pandemic (Ahmed et al., 2020; Lu et al., 2022a).

At the same time, this study found that after OESAG intervention, loneliness decreased significantly, which supports hypothesis 3. It is consistent with previous findings on the relationship between loneliness and social support in other populations (e.g., Bareket-Bojmel et al., 2021; Lu et al., 2022a). During the pandemic, prevention measures such as home quarantine, social distancing, and reduction of social activities changed the original way of life style of people and had a great impact on their original interpersonal relationships and social support (Gan et al., 2022). According to previous research results (Lu et al., 2022b), loneliness mediates the relationship between social support and stress response. And this study may also provide evidence that OESAG can increase social support, and reduces stress response through loneliness.

However, the shame decrease did not reach statistical significance after the OESAG intervention, which is inconsistent with hypothesis 4. This may be due to the more complex intervention mechanism of shame. In previous studies, shame-related groups often intervene through more subdivided aspects, such as individual self-efficacy (Zhang et al., 2020) or self-perception (Li et al., 2006), and require more sessions or more targeted intervention methods (Cassone et al., 2016). Regrettably, OESAG as a single-session group intervention does not have such subtle conditions and settings. There is also a possible reason that compared with shame, loneliness is more likely to be changed in this kind of short-term, emotion-support-focused group intervention (Sun et al., 2022). At the same time, the human interaction created during OESAG intervention is a way to alleviate loneliness. When it comes to shame, further work may be required (e.g., more sessions, longer time etc.) Finally, because the subject selection method limits participation to only healthy individuals, and the shame under this Pandemic may mainly come from being infected (fear of spreading it or stigma), so participants have relatively low levels of shame which lead to the non-significant result.

Deficiencies of the current study are listed as follows. Only self-reported data without diverse dimensional indexes were used to examine the effect of the intervention. The study mainly focused on uninfected people, excluding those infected and suspected infected, so there was a lack of intervention data on infected and suspected infected groups. Therefore, the exploration of the stress reaction and the intervention during the pandemic is not comprehensive enough.

Though verified, this OESAG model still has limitations. First of all, according to the feedback of group members, the limited communication time in the group sometimes caused insufficient sharing of emotions. Secondly, since OESAG is mainly based on online emotional companionship, other specific problems of the subjects may not be targeted enough, which led to the unsatisfied effect of negative emotions that need a long time to change (the intervention effect on shame is not obvious). Thirdly, due to the sudden outbreak of the COVID-19 pandemic, OESAG was created at a rapid pace, there are no systematic recruitment criteria, and variables are not well controlled in member selection. The participants were not a true cross-section of Chinese society as so many participants had graduate degrees. It is possible that OESAG was effective for them in the ways it was because it is a mode that is particularly attractive to those with graduate degrees. Finally, this study proved that the OESAG can increase PSS, and decrease loneliness, shame, and stress, but there is no longitudinal research to demonstrate OESAG works, so we could not tell the mechanism in detail for the causal relationship of OESAG. This can be a topic for our further research.

### Conclusion

The COVID-19 pandemic is still ongoing, the ensuing psychological stress has spawned many social problems, and there is still a large gap in the psychological service needs of the public. OESAG, although a single-session online intervention, can reduce stress and alleviate some obvious negative emotions (i.e., loneliness) during the pandemic, which has positive significance for how to deal with the stress caused by the pandemic effectively and efficiently and strengthen the social support and psychological assistance that people need during the pandemic.

## Data availability statement

The original contributions presented in the study are included in the article/supplementary material; further inquiries can be directed to the corresponding authors.

## Ethics statement

The studies involving human participants were reviewed and approved by Tsinghua University Ethics Committee (2021) LSD No. (23). The patients/participants provided their written informed consent to participate in this study.

## Author contributions

YZ designed the experiment and did the data analysis and wrote the article together with HH. HH wrote the article with YZ. DT gave very important guidance and theory viewpoint. XL initiated and designed the whole program and did this research mainly with YZ, HH, DT, XL, FF, and JP. FF sponsored this program and mentored XL and YZ. JP helped YZ with data analysis. All authors contributed to the article and approved the submitted version.

## Funding

This research was supported by Shenzhen-Hong Kong Institute of Brain Science (grant no. NYKFKT2020004), Key Laboratory of Youth Network Psychology and Behavior, Ministry of Education & Hubei Key Laboratory of Human Development and Mental Health (Central China Normal University; grant no. 2019B08), and General topic of ideological and political education for college students of Beijing Normal University in 2021 (BNUSZ2021YB01).

## Conflict of interest

The authors declare that the research was conducted in the absence of any commercial or financial relationships that could be construed as a potential conflict of interest.

## Publisher’s note

All claims expressed in this article are solely those of the authors and do not necessarily represent those of their affiliated organizations, or those of the publisher, the editors and the reviewers. Any product that may be evaluated in this article, or claim that may be made by its manufacturer, is not guaranteed or endorsed by the publisher.
